# Tubular Aggregate Myopathies: Genetic Heterogeneity and Diverse Clinical Features Converging on Calcium Dysregulation

**DOI:** 10.3390/cells15070635

**Published:** 2026-04-01

**Authors:** Matteo Serano, Federica Fiore, Vincenzo Sorrentino, Daniela Rossi

**Affiliations:** 1 Department of Molecular and Developmental Medicine, University of Siena, 53100 Siena, Italy; matteo.serano@unisi.it (M.S.); federica.fiore@unisi.it (F.F.); daniela.rossi@unisi.it (D.R.); 2 Program of Molecular Diagnosis of Rare Genetic Diseases, Azienda Ospedaliera Universitaria Senese, 53100 Siena, Italy

**Keywords:** tubular aggregate myopathy (TAM), calsequestrin1 (CASQ1), skeletal muscle contraction, myopathy

## Abstract

**Highlights:**

TAM is an inherited muscle disorder caused by *STIM1* and *ORAI1* mutations that disrupt calcium entry, leading to tubular aggregates and muscle dysfunction. Its overlap with Stormorken syndrome suggests a disease continuum. Although the genetics are known, mechanisms behind aggregate formation and multisystem features remain unclear, highlighting the need for deeper insight and targeted therapies.

**What are the main findings?**

- Genetics and Pathogenesis: Tubular aggregate myopathy (TAM) is an inherited skeletal muscle disorder caused mainly by *STIM1* and *ORAI1* mutations, while *CASQ1* and *RYR1* variants are less common. Its core mechanism is defective store-operated calcium entry (SOCE), leading to tubular aggregate formation, muscle dysfunction, and systemic features.
- Clinical Spectrum: TAM and Stormorken syndrome represent a continuum with shared mutations and overlapping manifestations. Clinical expression is highly heterogeneous in age of onset, severity, and extra-muscular involvement.

**What are the implications of the main findings?**

- Understanding and Identifying the Disease: Defining how SOCE dysfunction leads to tubular aggregates will clarify disease progression, while improved genotype–phenotype correlations can enhance diagnostic accuracy and patient stratification.
- Advancing Treatment Strategies: Targeting calcium-regulating pathways and uncovering additional molecular contributors or modifiers may pave the way for mechanism-based and personalized therapies.

**Abstract:**

Tubular aggregate myopathy (TAM) is a rare inherited muscle disorder characterized by the abnormal accumulation of tubular aggregates (TAs) within skeletal muscle fibers. These aggregates, composed of compacted sarcoplasmic reticulum (SR) tubules, are strongly linked to disturbances in calcium (Ca^2+^) homeostasis. Clinically, TAM manifests with slowly progressive proximal muscle weakness, exercise intolerance, cramps, and myalgia, frequently beginning in childhood and often present with elevated serum creatine kinase levels. These symptoms can also be associated with some additional disorders, such as thrombocytopathy, miosis, hypocalcemia, hyposplenism, and ichthyosis, thereby resulting in a clinical picture that overlaps with symptoms of Stormorken (STRMK) syndrome. Considerable heterogeneity exists in age of onset, severity, and extra-muscular involvement, suggesting that TAM and STRMK represent a continuum rather than distinct entities. Histopathological hallmarks include TAs staining positive for SR proteins and displaying a honeycomb-like ultrastructure, consistent with aberrant SR remodeling. Mutations in genes encoding key regulators of store-operated calcium entry (SOCE), including STIM1 and ORAI1 have been identified as major contributors to TAM and its broader clinical spectrum, which encompasses STRMK syndrome, whereas mutations in CASQ1 and RYR1, have been described in only a minority of patients. Despite advances in delineating the genetic and molecular basis of TAM, key questions remain regarding the mechanisms that drive TAs formation and translate Ca^2+^ dysregulation into muscle dysfunction and multisystem disease. Understanding the molecular mechanisms underlying TAM and STRMK syndrome is crucial for developing targeted therapies. Moreover, further research is needed to elucidate additional pathways involved in disease progression and to refine genotype–phenotype correlations. This review summarizes current knowledge on the genetics, pathophysiology, clinical features, and diagnostic hallmarks of TAM, with particular emphasis on the role of Ca^2+^ homeostasis.

## 1. Tubular Aggregates (TAs)

**Figure 1:**
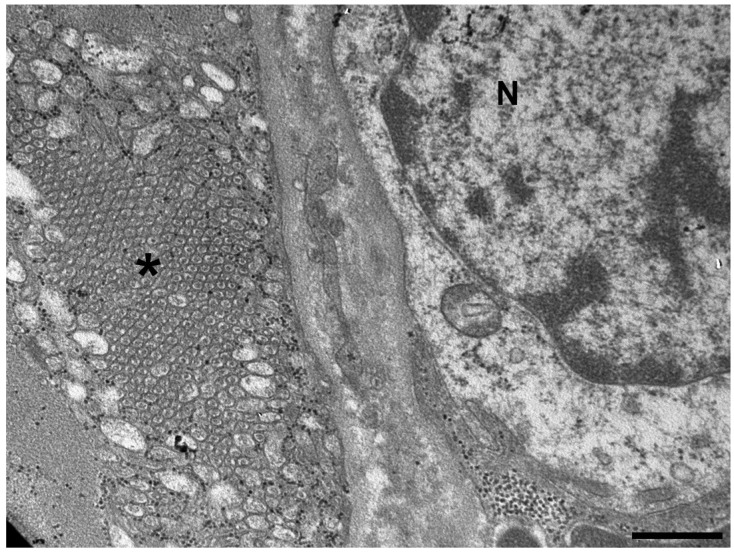
Representative transmission electron micrographs of a human muscle biopsy showing large tubular aggregates (N, nucleus; *, tubular aggregate). Bar: 500 nm [23].

Tubular aggregates (TAs) are the characteristic hallmark of TAM, they were described for the first time by Engel and colleagues in 1970 [1] as abnormally compacted membrane tubules derived from the sarcoplasmic reticulum (SR), which may or not contain dense material (Figure 1) [1,2,3,4,5]. The derivation of TAs from the SR was supported by the presence of SR proteins, including Sarcoplasmic/Endoplasmic Reticulum Ca^2+^-ATPase (SERCA) and Calsequestrin (CASQ) [4,6]. In cryosections, TAs appear as irregular bright red inclusions when using a modified Gomori trichrome technique and stain darkly with NADH-tetrazolium reductase (NADH-TR). In contrast, they remain negative for succinate dehydrogenase (SDH) staining [4,5,7], reflecting the absence of mitochondrial enzymatic activity within these structures and thereby helping to distinguish them from mitochondrial abnormalities. Immunohistochemically, TAs strongly react with antibodies, not only against SR proteins including SERCA1, stromal interaction molecule 1 (STIM1), CASQ1, and ryanodine receptor type 1 (RYR1), but also against some sarcolemmal proteins participating in Ca^2+^ signaling such as the Dihydropyridine Receptor (DHPR) and the Ca^2+^ release-activated calcium channel protein 1 (ORAI1). At the ultrastructural level, TAs appear as stacks of parallel straight tubules, arranged in a honeycomb-like pattern when observed in transverse sections [5,8]. 

In animal models, TAs accumulate preferentially in fast-twitch fibers of aging muscles [4,6,9], and their formation appears to be influenced by sex hormones, as they are reported to be absent in female’s muscles and reduced by estrogen treatment in aged male mice [10,11]. TAs have been also observed in mice carrying genetic alterations such as Dystrophin or/and Caveolin-1 and Caveolin-2 deficiency [12,13], as well as in rats exposed to extreme hypoxia [14]. Notably, long-term voluntary wheel running reduced TA accumulation in aged mice, suggesting that exercise can counteract their development [15].

In humans, the presence of TAs has been reported across unrelated neuromuscular disorders. Similarly to observations in mice, in human skeletal muscles, TAs are predominantly found in fast-twitch fibers (type II) and are only rarely observed in type I fibers [1,3,16,17,18,19,20]. The mechanisms underlying TA formation remain largely unclear; current models propose that they reflect SR membrane remodeling with protein misfolding/aggregation, likely promoted by impaired Ca^2+^ homeostasis, as a secondary non-specific response to cellular distressing conditions [4,6,21,22]. 

## 2. Tubular Aggregate Myopathies (TAMs)

Tubular aggregate myopathy (TAM) is a rare inherited muscle disorder characterized by the accumulation of TAs within skeletal muscle fibers. TAM predominantly affects the proximal muscles of the lower limbs, leading to gait disturbances and challenges in activities such as stair climbing, running, or rising from a squatting position. Common primary symptoms associated with TAM include progressive muscle weakness that are typically childhood-onset, mild to moderate in severity, and slowly progressive, as well as cramps, myalgia, mild hypocalcemia, and exercise intolerance [24,25,26,27,28,29]. Most patients also exhibit elevated serum creatine kinase (CK) levels, sometimes reaching up to ten times that of the normal upper limit. Elevated CK levels can be the earliest clinical sign of this condition [25]. A bleeding diathesis is also commonly observed, characterized by a mild bleeding tendency and thrombocytopathy [28,29]. Additionally, patients may present with hyposplenism or asplenia, indicating impaired or absent splenic function [27,28,29]. Less frequently, some individuals exhibit features such as short stature, mild intellectual disabilities including dyslexia, ichthyosis, headaches, and mild anemia [27,29,30,31,32].

**Figure 2:**
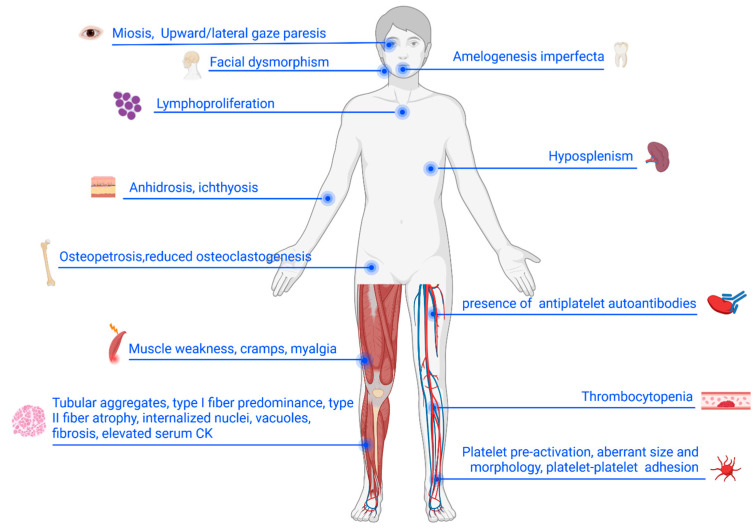
Multisystem manifestations of TAM and related disorders. Schematic overview of tissues and organs affected by TAM/STRMK. Clinical manifestations and pathological alterations are indicated in skeletal muscle, eye, skin, spleen, immune system, and other sites implicated in disease pathogenesis. Created in https://BioRender.com.

Several studies suggest that TAM overlaps clinically with other disorders, especially Stormorken (STRMK) syndrome and some forms of congenital myasthenic syndromes, underscoring the tight link between Ca^2+^ homeostasis and muscle pathophysiology [25,26,27,28,29,30,31,32,33]. This relationship reflects the fact that TAs originate from the SR, a key structure involved in intracellular Ca^2+^ handling; therefore, disturbances in Ca^2+^ regulation are thought to play a central role in their formation and in the associated muscle dysfunction. STRMK syndrome is a multisystemic disorder characterized by TAM-specific clinical manifestations occurring together with miosis, thrombocytopenia, ichthyosis, hyposplenism, dyslexia, and short stature [30]. TAM and STRMK are now considered part of a shared clinical spectrum, characterized by marked phenotypic heterogeneity [25,26,28]. In fact, clinical presentations range from patients displaying predominantly skeletal muscle involvement, with variable age of onset and severity of muscle weakness (i.e., patients diagnosed with TAM), to individuals exhibiting the full multisystemic manifestations of STRMK syndrome (Figure 2).

## 3. Molecular Genetics of TAM: STIM1 and ORAI1

Integrated clinical, histopathological, genetic, and functional studies identify dysregulation of intracellular Ca^2+^ homeostasis as a central pathogenic mechanism of TAM. Accordingly, patients with TAM/STRMK commonly carry heterozygous gain-of-function (GOF) mutations in *STIM1* or *ORAI1*, resulting in constitutive or dysregulated store-operated Ca^2+^ entry (SOCE) activation [29,32,34,35,36]. In contrast, recessive loss-of-function (LOF) mutations in the same genes cause a distinct clinical entity characterized by immunodeficiency and a comparatively mild myopathy, predominantly in the absence of TAs [37]. *STIM1* and *ORAI1* mutations span a broad clinical spectrum, ranging from early-onset, progressive muscle weakness with multisystem involvement to adult-onset myalgia or cramps without overt muscle weakness, highlighting the complexity of genotype–phenotype correlations in these disorders [2,25].

**Figure 3:**
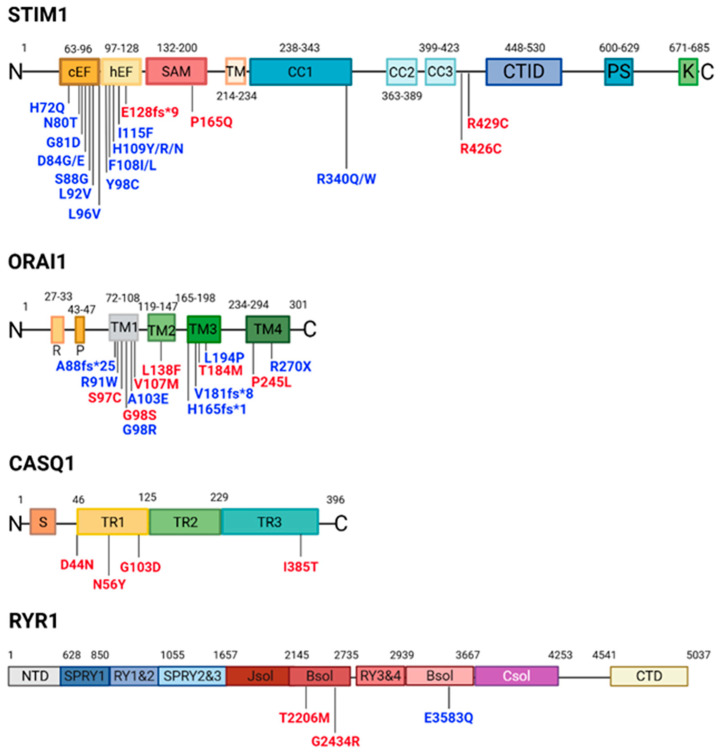
Schematic overview of STIM1, ORAI1, CASQ1, and RYR1 proteins. Distinct protein domains are highlighted in different colors. Gain-of-function mutations and loss-of-function mutations were respectively reported in blue and red. Created in https://BioRender.com. Abbreviations are as follows. STIM1 contains canonical EF-hand (cEF) motif, hidden EF-hand (hEF) motif, sterile a-motif (SAM), coiled-coil domains (CC1–CC3), a transmembrane domain (TM), a C-terminal inhibitory domain (CTID), a serine/proline-rich domain (PS), and a lysine-rich sequence (K) (see Ref. [38]). ORAI1 contains arginine- (R) and proline-rich (P) regions, as well as four transmembrane domains (M1–M4) (Adapted from Ref. [37]. Copyright year: 2020, copyright owner’s name: Silva Rojas et al.). Calsequestrin 1 (CASQ1) contains a signal peptide (S) and three similar domains characterized by a thioredoxin-like fold (TR1–TR3) (see Ref. [37]). RYR1 contains an N-terminal domain (NTD), three SPRY domains (SPRY1–3) with two tandem repeats (RY1 and RY2) located between SPRY1 and SPRY2, a junctional solenoid (J-Sol) domain, a bridging solenoid (B-Sol) domain, two tandem repeat domains (RY3 and RY4), and a central domain or core solenoid (C-Sol). Six transmembrane domains are positioned before the C-terminal domain (CTD) (see Ref. [39]).

In the following sections, the genes involved in TAM will be described, focusing on the structural and functional consequences of disease-causing variants in *STIM1*, *ORAI1*, and other genes involved in Ca^2+^ signaling in the skeletal muscle (see Figure 3).

### 3.1. STIM1

The stromal interaction molecule 1 (STIM1) gene comprises 12 exons and encodes a 685-amino-acid protein [40]. Alternative splicing of exon 11 generates the extended isoform STIM1-Long (STIM1L), which contains 791 residues due to an elongated C-terminal tail, while a less-characterized 540-amino-acid isoform, STIM1-short (STIM1S), is produced through an alternative splice event in exon 12 [40]. STIM1 is broadly expressed across tissues, whereas STIM1L shows enriched expression in the skeletal muscle and brain of rodents and predominantly in the skeletal muscle of humans [40,41].

STIM1 is a single-pass transmembrane protein with the N-terminal region residing in the ER/SR lumen and the C-terminal region oriented toward the cytosol [42,43]. The luminal region contains a canonical Ca^2+^-binding EF-hand, a non-canonical EF-hand, and a sterile α-motif (SAM) [44,45]. A fraction of STIM1 also localizes at the plasma membrane (PM) [46]. Three coiled-coil domains (CC1–CC3) are present in the C-terminal cytosolic region that follows the transmembrane domain (TM). The CC1 and CC2 domains contain the *STIM–Orai-activating region* (SOAR) also known as CAD (*CRAC activation domain*). Following the CC3 domain, there is the *C-terminal inhibitory domain* (CTID), which overlaps the CRAC modulatory domain. The most C-terminal region contains a serine/proline-rich domain (PS) and a lysine-rich sequence [45,47,48]. 

Upon endoplasmic reticulum (ER) Ca^2+^ depletion, the EF-hand and SAM domains unfold, triggering conformational rearrangements that promote CC1–CC1 dimerization, extension of the STIM1 cytosolic region, and exposure of the SOAR domain, thereby enabling its interaction with ORAI1. The poly-lysine region in the C-terminus contributes to membrane association and supports STIM1 clustering near the plasma membrane. Altogether, these structural changes trigger ORAI channel opening and initiate store-operated Ca^2+^ entry (SOCE) [45,49,50,51]. The role of SOCE and the function of ORAI channels will be discussed in more detail in the following paragraphs.

Upon endoplasmic reticulum (ER) Ca^2+^ depletion, Ca^2+^ dissociation from the EF-hands initiates conformational rearrangements that promote CC1-mediated dimerization, release of the SOAR and poly-lysine domains, and extension of activated STIM1 dimers toward the PM, where they can further oligomerize and bind ORAI1 channels, thereby triggering their opening and initiating store-operated Ca*^2+^* entry (SOCE) [38,45,49,52].

#### STIM1 Pathogenic Variants

To date, fourteen *STIM1* variants have been linked to TAM and STRMK syndrome. Among these, twelve variants cluster within the conventional and non-conventional EF-hand domains, while two variants have been identified in the cytosolic CC1 domain.

The most frequent variant is the R304W in the cytosolic CC1 domain, identified in twelve unrelated families from diverse geographical backgrounds [29,32,34,50,51,53]. Another variant affecting the same arginine residue, R304Q, has been reported in a single family [50]. Establishing a clear genotype–phenotype correlation remains challenging. The R304W variant has predominantly been associated with the full clinical spectrum of STRMK syndrome [21,29,34,50], although isolated cases presenting only with muscle weaknesses have also been documented [50]. All individuals carrying the R304Q mutation exhibited a consistently milder phenotype compared to R304W cases, with some patients remaining clinically asymptomatic [50].

Seven distinct variants (H72Q, N80T, G81D, D84E, D84G, S88G, L96V) have been identified within the canonical EF-hand motif [2,35,50,54,55], while five variants (F108I, F108L, H109N, H109R, I115F) affect the non-canonical EF-hand [2,26,35,53]. These variants are believed to either disrupt Ca^2+^ coordination or destabilize the interaction between the EF-hand motifs and the SAM domain (Figure 3). As a consequence, mutant STIM1 adopts an activated conformation even in the absence of ER Ca^2+^ depletion, leading to constitutive clustering of STIM1, persistent activation of ORAI1 channels, and increased SOCE. This gain-of-function mechanism results in chronic elevation of cytosolic Ca^2+^ levels and disruption of Ca^2+^ homeostasis, which is thought to underlie the pathogenesis of TAM.

Patients harboring EF-hand variants primarily presented with a heterogeneous myopathic phenotype, occasionally accompanied by isolated non-muscular features of STRMK syndrome. The N80T and L96V variants appear to be associated predominantly with myalgia, whereas the other EF-hand mutations were more frequently linked to proximal muscle weakness [2,26,35,53,54,55]. Notably, the substitution of the F108 residue resulted in divergent clinical presentations: the F108I mutation was associated with childhood-onset proximal muscle weakness, whereas F108L led to adult-onset myalgia [2,35].

Even the H109N variant, observed in two unrelated families, exhibited variable phenotypic expression. One family displayed predominant post-exercise fatigability and episodic diplopia, while the second family presented with lower-limb muscle weakness and contractures [2].

Magnetic resonance imaging analysis of five TAM patients carrying *STIM1* mutations (N80T, I115F, H109R, F108I) revealed a consistent pattern of lower-limb muscle involvement, characterized by atrophy and fat infiltration in the thigh and posterior lower leg, with relative sparing of the gracilis, tibialis anterior, and, to a lesser extent, the short head of the biceps femoris. Notably, involvement of the flexor hallucis longus, rarely affected in other myopathies, may represent a distinguishing feature of TAM/STRMK syndrome [56].

### 3.2. ORAI1

ORAI proteins are key components of Ca^2+^ release-activated Ca^2+^ (CRAC) channels, which are characterized by high Ca^2+^ selectivity and low conductance [57,58]. CRAC channels are activated following depletion of intracellular Ca^2+^ stores and are subsequently restrained by Ca^2+^-dependent inactivation (CDI) in response to local rises in cytosolic Ca^2+^ concentrations [59]. ORAI proteins are PM proteins composed of four α-helical transmembrane domains (M1–M4), with cytosolic N- and C-terminal regions and two extracellular and one intracellular connecting loop [49].

Structural studies, including crystallographic analyses of Drosophila melanogaster ORAI, revealed that functional ORAI channels assemble as hexamers. The ion-conducting pore is primarily formed by the M1 transmembrane helices, which confer Ca^2+^ selectivity via a conserved acidic glutamate ring at the channel entrance, whereas M2–M3 helices form a second ring that stabilizes and electrically isolates the pore, while the M4 helices constitute an outer ring interfacing with the PM [58,60,61,62,63,64]. The unusually low conductance of CRAC channels is attributed to a rigid hydrophobic segment within the central pore region [64].

Skeletal muscle expresses three ORAI isoforms (ORAI1, ORAI2, and ORAI3), with ORAI1 representing the principal pore-forming subunit of CRAC channels in this tissue [65].

ORAI1 activation is driven by direct coupling with STIM1, primarily via its C-terminal domain, with additional contributions from the N-terminal region and the intracellular loop linking M2 and M3 [49]. To prevent excessive Ca^2+^ influx, ORAI1-dependent SOCE is tightly regulated by two forms of Ca^2+^-dependent inactivation. Fast CDI occurs within milliseconds and is thought to result from Ca^2+^ binding to regulatory sites located in close proximity to the channel pore. In contrast, slow CDI develops over tens of seconds and involves additional regulatory mechanisms, including SOCE-associated proteins and the dynamic regulation of local cytosolic Ca^2+^ microdomains. In this context, mitochondria positioned near ER–PM junctions can take up Ca^2+^ via the mitochondrial Ca^2+^ uniporter, thereby buffering the rise in Ca^2+^ concentration in the vicinity of active ORAI1 channels. By shaping these local Ca^2+^ microdomains, mitochondrial Ca^2+^ uptake modulates the kinetics and extent of slow CDI [59].

#### ORAI1 Pathogenic Variants

To date, six distinct heterozygous missense variants in *ORAI1* have been identified in patients with TAM and STRMK syndrome, all affecting highly conserved amino acid residues within the transmembrane domains [22,25,34,65]. The mutations S97C, G98S, and V107M occur in the M1 domain, while L138F, T184M, and P245L alter residues located in the other transmembrane domains shaping the outer channel rings. Functional studies indicate that these ORAI1 mutations confer a gain-of-function phenotype by altering channel gating or pore architecture, thereby promoting constitutive Ca^2+^ influx independently of ER Ca^2+^ store depletion. This persistent activation of ORAI1 channels results in enhanced SOCE and chronic elevation of cytosolic Ca^2+^ levels, ultimately disrupting Ca^2+^ homeostasis in skeletal muscle fibers. The G98S variant has been reported in two unrelated families and affects a residue within the rigid pore region that limits ion conduction [66]. Both families presented severe phenotypes, characterized by early-onset muscle weakness and joint contractures [22,67]. Interestingly, a neighboring residue, S97, was found mutated in a family displaying a milder late-onset phenotype, with muscle weakness, cramps, and chronic elevation of CK levels [25].

Muscle cramping, stiffness, and joint contractures were consistently observed in most patients carrying *ORAI1* mutations [2,22,25,34,68] and appeared less frequently in those with *STIM1* mutations. Additionally, muscle weakness in *ORAI1*-related patients tended to be more diffuse, whereas *STIM1*-related muscle weakness was predominantly proximal. Non-skeletal muscle manifestations were generally milder in *ORAI1* patients and were limited to isolated features of STRMK syndrome, including miosis, dyslexia, and ichthyosis, with no reported cases of thrombocytopenia, asplenia, or short stature [2,25,34,67].

### 3.3. Impairment of SOCE in TAM

STIM1 and ORAI1 are core components of SOCE, a Ca^2+^-signaling mechanism that couples Ca^2+^ influx across the PM to the filling state of intracellular Ca^2+^ stores in both excitable and non-excitable cells [69,70,71]. Decades of research have established that SOCE is activated when STIM1 detects ER Ca^2+^ depletion and undergoes conformational rearrangements that drive its clustering at ER–PM junctions, where it directly activates ORAI1 [72,73,74,75,76]. The resulting Ca^2+^ influx supports cytosolic signaling while promoting refilling of ER Ca^2+^ stores [52,70,77,78,79].

In skeletal muscle, STIM1 is the principal SOCE regulator, whereas STIM2, characterized by lower Ca^2+^ affinity and slower ORAI1 activation, plays a more modulatory and comparatively less explored role [43,80]. Muscle fibers also express the long splice variant STIM1L, whose C-terminal extension promotes cortical actin association and constitutive pre-positioning near the PM, enabling the rapid SOCE kinetics required for repetitive Ca^2+^ cycling during sustained contraction [40,42]. Beyond ORAI1, both STIM1 and STIM1L have been shown to interact with transient receptor potential canonical (TRPC) channels, adding further layers of regulation to Ca^2+^ entry in muscle fibers [81]. 

Given the central role of STIM1-ORAI1 coupling in Ca^2+^ signaling, disease-causing variants in either protein can perturb SOCE, thereby disrupting Ca^2+^ homeostasis and contributing to human pathologies [82,83].

#### Possible Mechanisms Involved in Biogenesis of TAs

In TAM, Ca^2+^ homeostasis is disrupted, typically by pathogenic variants that alter Ca^2+^ entry pathways and promote abnormal intracellular Ca^2+^ signaling [24,84]. However, how disease-causing variants in *STIM1* and *ORAI1* converge on TA formation and muscle dysfunction remains incompletely defined. TAs are widely viewed as the morphological endpoint of progressive SR remodeling and compaction, rather than a *de novo* structure. A prevailing mechanistic model proposes that chronic stress, particularly sustained Ca^2+^ dyshomeostasis with secondary proteostasis impairment, induces SR “distress” and progressive reshaping/aggregation of SR membranes into increasingly ordered arrays [5]. Longitudinal studies in aging fast-twitch fibers further support a stepwise *in vivo* biogenesis, with marked dependence on age, sex, and fiber type [9,85]. Interestingly, *STIM1* GOF TAM models display features like weakness, reduced muscle mass, and mitochondrial alterations, yet do not develop overt TAs. This contrasts with ORAI1 GOF models, in which TAs are observed, suggesting that additional species-, age-, or context-dependent factors may contribute to TA biogenesis [86].

Across disease models, SOCE perturbation and Ca^2+^ imbalance have been linked to downstream pathways such as mitochondrial dysfunction and ER stress/unfolded protein response, which may progressively contribute to myofiber pathology over time [86,87,88]. 

Consistent with a stress-driven process, experimental mitochondrial/energetic perturbation (ATP synthase inhibition) can trigger SR stress responses and TA development, highlighting mitochondria–SR crosstalk as a plausible upstream driver of SR remodeling [89]. Importantly, TA burden is not fixed: long-term endurance exercise reduces TA formation in aged muscle and limits aberrant STIM1/ORAI1 accumulation within TAs, indicating that TA biogenesis is modifiable and influenced by activity-dependent Ca^2+^ handling demands [15]. This effect is generally considered beneficial, as the reduction in TA content is accompanied by improved Ca^2+^ handling and a greater ability of muscle fibers to utilize extracellular Ca^2+^. However, it remains unclear whether TAs arise primarily as a consequence of altered Ca^2+^ homeostasis or whether they themselves contribute to muscle dysfunction. Overall, despite substantial clinical and histopathological variability, the available evidence supports altered Ca^2+^ homeostasis as a central event linking defects in SOCE and Excitation Contraction coupling to SR remodeling and TA formation.

## 4. Molecular Genetics of TAM: CASQ1 and RYR1

### 4.1. CASQ1

In addition to causative variants in *STIM1* and *ORAI1*, TAM has also been recently associated with variants in *CASQ1* [90,91]. CASQ1 is an acidic, high-capacity Ca^2+^-binding protein of the SR, predominantly expressed in fast-twitch muscles [92,93,94]. Structural studies indicate that CASQ1 undergoes Ca^2+^-dependent polymerization within the SR lumen, a property that underlies its ability to support high-capacity Ca^2+^ storage [95,96,97,98,99,100].

Functionally, CASQ1 helps maintain high concentrations of Ca^2+^ in the SR, from which it is released following activation of RYR1 channels during the excitation–contraction (EC) coupling mechanism in order to activate muscle contraction. CASQ1 also modulates RYR1 activity through interactions within the RYR1–Triadin–Junctin complex [101,102,103,104,105]. In addition, upon SR depletion, CASQ1 has been proposed to bind STIM1 and limit STIM1-ORAI1 coupling, thereby reducing Ca^2+^ influx across the PM via SOCE [106,107,108]. Additionally, CASQ1 knockout mouse models exhibit constitutive remodeling of the SR, leading to the formation of specialized junctional structures known as calcium entry units (CEUs), which promote sustained SOCE activation [109,110].

#### CASQ1 Pathogenic Variants

About ten years ago, *CASQ1* was identified as the third gene associated with TAM. To date, multiple missense variants in *CASQ1* have been reported in TAM, including D44N, N56Y; G103D, and I385T [90,91]. In particular, G103D is the only recurrent mutation found in two unrelated cases [90,91].

Clinically, *CASQ1*-related TAM is typically milder than *STIM1/ORAI1*-associated cases, presenting with slowly progressive adult-onset weakness or exercise-induced fatigue/myalgia, predominantly in the lower limbs and without prominent extramuscular features [90,91]. This likely reflects the skeletal-muscle-restricted expression of *CASQ1*, which is enriched in type II fibers; accordingly, TAs are detected predominantly/exclusively in type II fibers and stain for SERCA1, the type II fiber Ca^2+^ pump [90,91,94]. Functionally, all tested mutant CASQ1 proteins show reduced SR Ca^2+^ storage capacity, but only a subset of these variants enhances SOCE, suggesting that muscle pathology may also involve SOCE-independent mechanisms and activation of additional downstream pathways [91,111].

### 4.2. RYR1

The identification of *RYR1* mutations in TAM further supports the concept that TAs may represent a common downstream structural response to diverse disturbances in Ca^2+^ signaling [23]. *RYR1* encodes the ryanodine receptor type 1 (RYR1), the principal Ca^2+^ release channel of the SR in skeletal muscle, localized in the terminal cisternae of the SR. Upon depolarization, RYR1 channels are activated to rapidly release Ca^2+^ from the SR lumen to trigger muscle contraction [112,113].

RYR1 is a large homotetrameric channel embedded in a macromolecular complex of regulatory proteins and is modulated by cytosolic and luminal cues (e.g., Ca^2+^/Mg^2+^, ATP, calmodulin, FKBP12, and post-translational modifications) that tune channel open probability across functional states [39,114,115,116,117,118,119].

On the luminal side, RYR1 is functionally coupled to CASQ via Triadin and Junctin, allowing SR Ca^2+^ load to influence release and helping prevent excessive depletion during sustained activity [120,121]. Beyond EC coupling, RYR1 has also been linked to SOCE regulation: RYR1-dependent SR Ca^2+^ dynamics may shape local microdomains that influence STIM1-ORAI1 activation at junctions closely associated with triads [122,123].

#### RYR1 Pathogenic Variants

RYR1 variants were first linked to Malignant Hyperthermia Susceptibility (MHS), a pharmacogenetic condition triggered by halogenated anesthetics [124], and later to central core disease (CCD) and other *RYR1*-related myopathies (RYR1-RM) [125,126]. More recently, two dominant *RYR1* variants, T2206M and G2434R, previously linked to MHS, have been identified in two unrelated individuals affected by TAM, who tested negative for mutations in *STIM1*, *ORAI1*, and *CASQ1* [23,124,125,126,127]. This finding was unexpected, as RYR1-RM are typically associated with core-type alterations in muscle, rather than TAs [124,125,126,127,128,129,130]. Both patients exhibited mildly elevated serum CK and reported episodic muscle stiffness, often triggered by repetitive exertion or cold exposure symptoms not classically associated with TAM. The clinical presentation in these two patients was milder than that observed in most TAM cases linked to *STIM1* or *ORAI1* variants, raising the possibility that RYR1-associated TAM may be underdiagnosed [23].

## 5. TAM Mouse Models

Currently available mouse models of TAM primarily focus on SOCE dysregulation pathways and can be broadly classified into three main categories: *Stim1* GOF knock-in lines, *Orai1* GOF knock-in lines, and LOF *Casq1* (knockout) models used to interrogate TAM-associated mechanisms. STIM1 GOF models (e.g., *Stim1*^R304W/+^ and the luminal EF-hand *Stim1*^I115F/+^ knock-in) reproduce SOCE overactivation and several systemic and muscle-relevant consequences, making them valuable for mechanistic dissection and therapeutic testing. However, a key limitation in these models is that TAs are typically absent, indicating either species- or context-dependent requirements for TAs biogenesis, limiting their utility for studying TAs formation *per se* [83,131].

In contrast, *Orai1* GOF knock-in models based on TAM/STRMK alleles variants found in patients (e.g., *Orai1*^G100S/+^ and *Orai1*^V109M/+^) show higher histopathological face validity because they develop TAs, providing a more direct *in vivo* model to study TAs biogenesis and its relationship to altered Ca^2+^ entry. Their main caveats are developmental/age-dependent compensation and allele-specific systemic involvement that can influence penetrance and experimental endpoints [24,132,133].

Finally, *Casq1-null* (LOF) mice do not model TAM genetics directly, but they provide a powerful model to probe how TAM-associated *CASQ1* variants alter SR Ca^2+^ storage and SOCE regulation in the native tissue context. Indeed, in this model, the compromised ability to store Ca^2+^ in the SR triggers adaptative mechanisms, including constitutive activation of the SOCE. A characteristic of this model is that Casq1 ablation drives also a broad SR/triad remodeling characterized by SR membrane reorganization into stacked cisternae closely opposed to remodeled T-tubules [134,135]. These specialized junctions, termed calcium entry units (CEUs), are triad-like SR–T-tubule contacts that concentrate Stim1/Orai1 machinery to support sustained SOCE and facilitate store refilling during prolonged muscle activity [109,110].

The main mouse models currently available to study TAM are summarized in Table 1, detailing the corresponding mutation, associated skeletal muscle phenotype, and the proposed pathogenic mechanisms. Overall, these models are not exhaustive: they capture key genetic drivers and Ca^2+^-signaling mechanisms of TAM, but they do not fully represent the breadth of human disease (allelic heterogeneity, variable penetrance, age of onset, and modifying factors). On the other hand, they are highly informative for testing causality and downstream pathways of SOCE/SR dysfunction, yet they only partially allow a deep dissection of TA biogenesis because TA formation is allele-, age-, muscle-, and species-dependent (e.g., several *Stim1* GOF lines show robust disease phenotypes but little/no TAs, whereas some *Orai1* GOF models do form TAs).

Key limitations include developmental effects, compensatory remodeling of Ca^2+^ entry with age, differences in fiber-type composition and lifespan between mice and humans, and the possibility that TAs require additional contributing factors (e.g., metabolic stress, hormonal context, activity level) that are not uniformly present in standard housing conditions. In terms of clinical translation, the models can reproduce important aspects of the patient spectrum (muscle weakness/fatigue and, in some alleles, multisystem involvement), but they generally do not replicate the full variability seen in patients and may over- or under-estimate specific features dependent on the mutation class and genetic background.

Availability of mouse models has provided a tool to move forward a therapeutic intervention in TAM. Indeed, given the central role of SOCE in maintaining cellular Ca^2+^ homeostasis by refilling Ca^2+^ stores in almost all cell types, it is not surprising that STIM1 and ORAI1 have been the focus of numerous studies aimed at targeting them, as well as their associated regulatory proteins, to modulate cellular functions in both normal and tumorigenic cell proliferation. Since ORAI1 is accessible to extracellular compounds, it represents a particularly attractive therapeutic target, potentially even more than STIM1. Unfortunately, although many inhibitors have been identified, only a few of them have demonstrated promising drug-like properties to date [41,136,137,138,139,140,141]. Nevertheless, these studies, regardless of the original cellular context in which they were conducted, may yield compounds that target ORAI1 or STIM1 and could also be beneficial for patients with TAM and/or STRMK syndrome.

**Table 1:** Currently available mouse models for study TAM and TA development: genotype, key phenotype readouts, and altered mechanisms.

Gene	Mutation	Type	Functional Effect	Phenotype	Proposed Mechanism
STIM1	R304W c.910C>T	Heterozygous, missense	GOF	-Homozygous: embryonic lethal. -Heterozygous: Reduced survival/growth with splenomegaly, skeletal abnormalities, elevated serum CK and resting Ca^2+^; weakness with delayed kinetics/partial airway obstruction; myopathy with internal nuclei/fibrosis/inflammation, type I shift, mitochondrial swelling. Do not present TAs [86].	Constitutive activation of ORAI1 channels due to exposure of the SOAR domain and impaired Ca^2+^-dependent inactivation.
	I115F c.343A>T	Heterozygous, missense	GOF	-Mice are viable and fertile, no early mortality reported. -Phenotype: Reduced body weight and age-dependent kyphosis and tremors. Increased SOCE from 1 month of age (basal cytosolic Ca^2+^ normal); progressive myopathy (reduced muscle mass and fiber CSA, necrosis, regeneration, centralized nuclei, and fibrosis) with mitochondrial abnormalities. Motor coordination and endurance are impaired, while grip strength is largely preserved. -Pseudo-aggregates are present (no true TAs) [131].	Altered EF-hand Ca^2+^ sensing in STIM1 increases SOCE activity, leading to chronic Ca^2+^ dysregulation. Sustained Ca^2+^ influx promotes mitochondrial dysfunction and progressive myofiber damage, driving muscle wasting and functional decline.
	D84G p.Asp84Gly	Heterozygous, missense	GOF	-Phenotype: atrophy/weakness with sarcopenia-like nuclear defects; resting Ca^2+^ and SR stores largely preserved (compensatory sarcolipin/Casq changes); defective nuclear Ca^2+^ transfer and broad transcriptional remodeling; macrothrombocytopenia/bleeding. -Constitutive SOCE activation. Rare TAs [35,142].	Loss of EF-hand Ca^2+^ sensing in STIM1, leading to constitutive SOCE and altered Ca^2+^ signaling, combined with a SOCE-independent role in regulating nuclear Ca^2+^ flux, nuclear envelope integrity, chromatin organization, and genomic stability, ultimately driving muscle atrophy and dysfunction.
	STIM1 -/-	Deletion	LOF	-Marked perinatal lethality and postnatal growth failure. -Phenotype: Low body mass, normal fiber number but reduced CSA and myonuclei (impaired myonuclear accretion), severe ultrastructural/mitochondrial damage. -Blunted ERK/AKT/p38 responses and calcineurin–NFAT dysregulation, with downregulation of metabolic/differentiation genes [143].	Loss of STIM1 abolishes SOCE, impairing Ca^2+^-dependent signaling pathways, which resulted in defective postnatal muscle growth, metabolic programming, mitochondrial integrity, and reduced muscle survival.
ORAI1	G100S	Heterozygous, missense	GOF	-Homozygous: embryonic lethal. -Heterozygous: Weakness and exercise intolerance with hypocalcemia and increased CK, minimal overt degeneration; prominent mitochondrial proteomic/functional defects with limited “classic” myopathic hallmarks. Age-dependent TAs (mainly in fast-twitch muscles detectable ~from 8 months) [67,133].	GOF mutation in the ORAI1 pore region causes constitutive, STIM1-independent SOCE, leading to chronic Ca^2+^ influx, mitochondrial dysfunction, as well as age-dependent TAs formation in the skeletal muscle.
	V109M	Heterozygous, missense	GOF	-Phenotype: Weakness and muscle atrophy, internal nuclei, increased resting Ca^2+^, short stature, splenomegaly and thrombocytopenia (some sex-specific traits). Moderate hypocalcemia/thrombocytopenia. Presence of Tas [32,91,132].	GOF mutation in the ORAI1 pore-forming domain causing loss of Ca^2+^ selectivity and increased cation permeability, leading to elevated basal Ca^2+^ influx, chronic Ca^2+^ overload, myofiber degeneration, and TAs formation.
	cORAI1 -/-	Deletion	LOF	-Muscle-specific Orai1 knockouts mice are viable with normal early postnatal development, unlike global knockout. Phenotype: Progressive atrophy (predominatli in soleus) with loss of type I fibers and severe type I CSA reduction. SOCE impaired with reduced SR Ca^2+^ content and Casq1 (no Casq2 compensation); force deficit at high frequency and increased fatigability; reduced grip/endurance; premature-aging traits; CEU remodeling (SR stacks without matching T-tubule elongation). TAs are absent [144,145].	Loss of ORAI1 impairs SR Ca^2+^ refilling and Ca^2+^-dependent signaling pathways, resulting in altered muscle fiber specification, preferential loss of oxidative fibers, mitochondrial dysfunction, defective CEU assembly, progressive muscle atrophy, and premature muscle aging.
CASQ1	CASQ1 -/-	Deletion	LOF	Phenotype: Progressive fast-twitch-predominant myopathy with reduced strength and impaired contractility, altered EC coupling, and fatigability. With aging: mitochondrial damage/oxidative stress, structural remodeling with core-like areas. High susceptibility to exercise/heat-triggered hyperthermic (malignant hyperthermia–like) crises. -Do not present TAs [122,135,146,147].	Loss of CASQ1 eliminates SR Ca^2+^ buffering, causing chronic SR Ca^2+^ depletion and destabilization of Ryr1-mediated Ca^2+^ release. Persistent store depletion promotes sustained activation of STIM1–ORAI1-dependent SOCE, leading to Ca^2+^ overload, oxidative stress, mitochondrial dysfunction, and pathological SR remodeling that drives core formation.

## 6. Conclusions

Taken together, this review outlines the clinical features of tubular aggregate myopathy (TAM) and the potential mechanisms underlying this inherited skeletal muscle disorder, caused primarily by mutations in *STIM1* and *ORAI1*, and, more rarely, in *CASQ1* and *RYR1*. These mutations alter SOCE regulation, leading to tubular aggregate formation and muscle dysfunction. Notably, *STIM1* and *ORAI1* mutations are also observed in patients with STRMK, highlighting a spectrum of overlapping phenotypes rather than entirely distinct disorders.

Taken together, TAM exemplifies how defects in calcium signaling can reshape muscle structure and function, with STIM1- and ORAI1-related SOCE dysfunction at the core of disease pathogenesis. However, although significant progress has been made in understanding TAM’s molecular basis, critical questions remain regarding the mechanisms driving aggregate formation and multisystem involvement. Addressing these unresolved mechanisms is essential for refining diagnosis and ultimately guiding the development of targeted, mechanism-based therapies.

## Data Availability

No new data were created or analyzed in this study.

## Abbreviations

The following abbreviations are used in this manuscript:

Ca^2+^	Calcium
CASQ	Calsequestrin
CK	Creatine Kinase
DHPR	Dihydropyridine Receptor
GOF	Gain of function
LOF	Loss of function
RYR1	Ryanodine receptor type 1
SERCA	Sarcoplasmic/Endoplasmic Reticulum Ca^2+^-ATPase
SOCE	Store-operated calcium entry
SR	Sarcoplasmic reticulum
STIM1	Stromal interaction molecule 1
STRMK	Stormorken
TA	Tubular aggregate
TAM	Tubular aggregate myopathy
